# Barriers to compliance with evidence-based guidelines for ventilator-associated pneumonia among critical care nurses: A scoping review

**DOI:** 10.12688/f1000research.128144.2

**Published:** 2023-03-24

**Authors:** Muna Al-Tamimi, Fatma Refaat, Wegdan Bani Issa

**Affiliations:** 1 Department of Nursing, College of Health Sciences, University of Sharjah, Sharjah, Sharjah, 27272, United Arab Emirates

**Keywords:** Barriers; Compliance, Critical care nurses; Ventilator-associated pneumonia; Prevention guidelines

## Abstract

**Background:** Healthcare organizations provide evidence-based guidelines designed to support nurses in preventing ventilator-associated pneumonia (VAP) in intensive care units (ICUs), but there are barriers to compliance with such guidelines. This review explicitly explored evidence of compliance barriers among critical care nurses.

**Methods:** A systematic search was conducted in PubMed, Cumulative Index to Nursing and Allied Health Literature (CINAHL), and EBSCO databases for relevant English-language studies published between January 2003 and June 2022, focused on barriers to nursing compliance with VAP prevention guidelines. Data was reported according to the Reporting Items for Systematic Reviews and Meta-Analyses extension for Scoping Reviews (PRISMAScR) guidelines.

Results:

230 publications were screened, resulting in 53 full-text articles being retrieved after removing duplicates, of which 13 relevant to the aims of the review and meeting the inclusion criteria were included for data extraction. One was a qualitative study, while the remainder were quantitative. Simple descriptive content analysis identified the barriers to critical care nurses’ compliance with VAP prevention guidelines, and categorized them as: (1) work environment barriers *(e.g*., *lack of equipment and supplies*; *lack of staff and time; lack of educational support*; and *ineffective supportive system*); (2) nurse-related barriers ( *limited personal competencies*); and (3) situation-related barriers ( *patient health, discomfort, and adverse events*).

**Conclusions:** This review revealed important evidence on barriers to VAP prevention guidelines compliance. Nurses are challenged mainly by work-environmental barriers along, with the presence of nurse and situational barriers. It is evident from the findings that further qualitative and mixed-methodology follow-up studies are recommended to further explore the issues in depth.  Healthcare leaders must be aware of these barriers and integrate work policies that assist in overcoming them, to increase compliance.

## Introduction

Ventilator-associated pneumonia (VAP) is a common and arduous respiratory infection that targets mechanically ventilated patients (MVPs) in intensive care units (ICUs) ( Hunter, 2012; Metersky & Kalil, 2018; Valles *et al.*, 1995). VAP is characterized as an infection of the pulmonary parenchyma that appears after at least 48 hours of endotracheal intubation for mechanical ventilation, with day 1 being the day of ventilator insertion. It causes about a third of nosocomial pneumonia cases in ICUs ( Torres *et al.*, 2017). According to Centers for Disease Control and Prevention (CDC) report, pneumonia was the most common infection in US acute care hospitals in 2015, with 32% occurring as a result of the use of ventilators ( Magill *et al.*, 2018). Another study conducted across three Gulf Cooperation Council countries (Bahrain, Oman, and Saudi Arabia) estimated that the local VAP rate was 217% higher than in the US, according to National Healthcare Safety Network data ( El-Saed *et al.*, 2016). Ventilated patients who develop VAP suffer higher mortality than ventilated patients who do not ( Ibrahim *et al.*, 2001; Safdar *et al.*, 2005). Moreover, VAP substantially increases ventilator time and length of ICU stay ( Papazian *et al.*, 2020), along with ICU care costs ( Warren *et al.*, 2003; Zimlichman *et al.*, 2013).

As a result of the high mortality, morbidity, and hospital expenses associated with VAP, a number of guidelines have been published since the 1980s to prevent and control it ( Rello *et al.*, 2002; Society & America, 2005). Efforts continued by health organizations and societies such as the European Centre for Disease Prevention and Control (ECDC) and the CDC to set and update evidence-based guidelines (EBGs) and strategies that markedly proved to minimize the occurrence of VAP and improve outcomes, and the quality of care (QoC) delivered to MVPs ( (IHI). 2012; Klompas *et al.*, 2022; Torres *et al.*, 2017).

Nurses play a pivotal role in providing safe and direct care to MVPs, including practicing VAP prevention strategies that are essential to prevent infection and therefore improve the quality of patient care and outcomes in ICUs ( Jansson *et al.*, 2013; Osti *et al.*, 2017). According to Pogorzelska *et al.* (2011), nursing compliance with VAP prevention guidelines in ICUs (hereinafter “NC”) should be at least 95%, in order to effectively reduce VAP incidence. Therefore, strict compliance with strategies and recommendations is required from nurses, who must appropriately perform several interventions and procedures to achieve optimum outcomes ( Miranda da Cruz & da Silva Martins, 2019; Rello *et al.*, 2013; Tabaeian *et al.*, 2017). Facilitating and promoting changes in patient care, and encouraging NC is crucial for VAP prevention success, and also providing regular feedback on process measure performance and outcome rates are among the best practices to facilitate adherence with guidelines ( Crunden *et al.*, 2005; Klompas, 2017).

According to Pogorzelska *et al.* (2011), nursing compliance with VAP prevention guidelines in ICUs (hereinafter “NC”) should be at least 95%, in order to effectively reduce VAP incidence. Therefore, strict compliance with strategies and recommendations is required from nurses, who must appropriately perform several interventions and procedures to achieve optimum outcomes ( Miranda da Cruz & da Silva Martins, 2019; Rello *et al.*, 2013; Tabaeian *et al.*, 2017).

Facilitating and promoting changes in patient care, and encouraging NC is crucial for VAP prevention success, and also providing regular feedback on process measure performance and outcome rates are among the best practices to facilitate adherence with guidelines ( Crunden *et al.*, 2005; Klompas, 2017).

In spite of the existence of clinical practice guidelines for preventing VAP, these guidelines are not consistently followed ( Cason *et al.*, 2007; Ricart *et al.*, 2003). Reported levels of compliance with and proper use of strategies vary widely in various contexts, across health systems, specialties, and nurses, ranging from 20% to nearly 100% ( Beattie *et al.*, 2012; Bird *et al.*, 2010; Rello *et al.*, 2002). Several studies showed that nurses generally exhibit low NC, which could be attributable to diverse factors ( Aloush *et al.*, 2018; Aloush & Al-Rawajfa, 2020; Jahansefat *et al.*, 2016; Jam *et al.*, 2018). Given the critical impact of NC on patients’ QoC, it is essential to identify the factors influencing it that might impede the proper implementation of EBGs, and hence affect patient outcomes.

There are few existing reviews of studies reporting NC barriers (hereinafter “NCBs”). Consequently, it is vital to comprehensively map the evidence relating to the findings available in this topic, how studies have been conducted, the key characteristics of studies, and important knowledge gaps. A scoping review is used to explore the breadth or extent of the literature, map and summarize the evidence, and inform future research ( Tricco *et al.*, 2016). This scoping review aimed to show the available evidence of the barriers toward critical care nurses’ (CCNs) NC. The primary objective of this scoping review is to understand the types and extent of evidence available in relation to NCBs. The detailed aims of this review were to: (1) examine the characteristics of studies that have reported the barriers toward preventing VAP guidelines among CCNs; (2) identify and summarize key findings of related studies; and (3) identify gaps of extant studies that may help inform future research in this area.

## Methods

### Design

The scoping review was conducted in accordance with the Joanna Briggs Institute (JBI) methodology to determine CCNs’ NCBs for VAP EBGs. It followed the frameworks of Arksey and O'Malley (2005) and Levac *et al.* (2010) that have underpinned the development of the JBI approach for conducting scoping reviews ( Peters *et al.*, 2015). Moreover, It is reported according to the Preferred Reporting Items for Systematic Reviews and Meta-Analyses extension for Scoping Reviews (PRISMA-ScR) to provide a guidelines to the review ( Tricco *et al.*, 2018). Population-Concept-Context (PCC) elements is followed in line with JBI’s recommendations for scoping reviews, in order to guide the inclusion criteria development, facilitate literature searches, and offer a robust framework of this scoping review ( Peters *et al.*, 2020).

### Review question

This scoping review examined the following research question: What are the barriers to compliance with the VAP prevention guidelines among CCNs. The population comprised nurses practicing in any country. The core concept examined was the barriers to compliance with VAP guidelines prevention and the context was ICUs.

### Eligibility criteria

The search was performed between March-June 2022 to include relevant studies in English language only (excluding non-English language studies as they required translation), using the electronic research databases Cumulative Index to Nursing and Allied Health Literature (CINAHL), PubMed, and EBSCO. Database searches were targeted to full-text, peer-reviewed articles, including primary research and any type of review. Relevant articles selected for review were published between January 2003 and June 2022, to include studies since the issuance of the CDC’s updated recommendation “Guidelines for Preventing Health-Care--Associated Pneumonia” ( Tablan *et al.*, 2003). Data were collected using the main keywords on VAP, Prevention guidelines, Barriers, Compliance, Intensive care, and Nurses. The detailed search strategy is available as additional file in the data availability. The participants of the included studies had to be CCNs and charge nurses who had worked directly with MVPs, with responsibility for implementing the VAP prevention guidelines (hereinafter “VAP PGs”) for which they were reporting the perceived NCBs. Assistant nurses, nurse managers and other health professionals were excluded. The focus of studies had to be identifying barriers to NC with VAP PGs. Other factors that were irrelevant or which did not hinder NC were excluded. The included studies had to have been conducted in ICUs using mechanical ventilation for patients. Table 1 summarizes the inclusion and exclusion criteria for the selected studies.

**Table 1.** Inclusion and exclusion criteria for selected studies.

Inclusion criteria	Exclusion criteria
English	Non-English
January 2003 and June 2022	Before or after the inclusion date
Primary studies and reviews	Theses, reports, editorials
Full text, peer-reviewed studies	Non-peer-reviewed studies, not available in full-text version, unpublished (grey) literature
Critical care nurses, including nurses in charge, directly responsible for mechanically ventilated patients, and applying strategies and guidelines for VAP prevention	Nurses in charge and nurse managers who do not care directly with mechanically ventilated patients. Assistant nurses, other healthcare professionals
Barriers (obstacles) to nurses’ compliance with VAP prevention guidelines	Other factors that irrelevant or not hindering nurses’ compliance with VAP prevention guidelines
ICUs caring for mechanically ventilated patients	

### Study selection

The search results were collated, uploaded to EndNote X9.3.3/2008 (RRID:SCR_014001; Clarivate Analytics, PA, USA), and duplicates were removed. Three reviewers (MA, FR, and WB) independently screened titles and abstracts to determine whether they met inclusion criteria. Relevant sources were retrieved in full. A hand search of the bibliographies of initially included studies was undertaken in order to identify additional relevant works not gleaned from the database search ( Peters *et al.*, 2020). All reviewers independently examined the full texts of selected citations to ensure that they met the inclusion criteria, and evidence sources that did not meet the inclusion criteria were excluded. During the selection process, disagreements between the reviewers were resolved through discussion, as detailed in the Results section.

### Data extraction

Data were extracted from the articles by the reviewers using JBI data extraction tool. Detailed information about the authors, years of publications, country, study aims, study methods, and key findings relevant to the review aim/s were extracted ( Table 2). The data extraction tool was revised and modified by all authors as necessary during the process of extracting data from each evidence source included in this review. A discussion was used to resolve disagreements between reviewers. There was no need to contact the authors of the articles due to the comprehensiveness of reported data ( *i.e.*, no missing data).

**Table 2.** Extraction sheet of the studies.

Author(s), year, country	Aim/purpose	Sample size	Methodology	Key relevant findings
Aloush and Al-Rawajfa (2020) Jordan	To evaluate the compliance of Jordanian nurses with VAP prevention guidelines and the barriers to compliance	294	Quantitative cross-sectional design Descriptive survey Self-administered questionnaire	Lack of education Lack of policies and protocols Lack of resources Shortage of staff
Aloush *et al.* (2018) Middle East (Jordan, Egypt, Saudi Arabia)	To investigate barriers and factors that affect nurses’ level of compliance with VAP prevention guidelines	471	Quantitative cross-sectional design Self-reported survey	Lack of education Lack of guidance Lack of a professional model Poor integration of research findings in practice Lack of experience Unavailability of resources
Al-Sayaghi (2021) Saudi Arabia	To determine the factors that affect nurses’ compliance and explore barriers faced by them	229	Quantitative cross-sectional design Descriptive survey Self-reported questionnaire	Shortage of nursing staff Lack of time Forgetfulness Hospital cost control policies Lack of continuous education Fear of unpredictable adverse effects and undesirable patient outcomes
Atashi *et al.* (2018) Iran	To explore barriers to VAP prevention	23	Qualitative descriptive study Semi-structured interviews	Inadequate or inappropriate equipment Heavy workload Staff shortage Inadequate staff training and supervision Limited professional competence Unfavorable environmental conditions Passive human resource management Unfavorable professional attitudes Low job motivation Limited professional accountability Nonstandard physical structure
Bankanie *et al.* (2021) Tanzania	To explore barriers to VAP prevention EBGs	116	Quantitative cross-sectional design Descriptive survey Self-administered questionnaire	Lack of skills Lack of staff Lack of knowledge Job discretion VAP procedures considered unnecessary
Dehghan *et al.* (2022) Iran	To examine registered nurses’ perceived barriers towards VAP prevention in southeast Iran	321	Quantitative cross-sectional design Descriptive survey Self-administered questionnaire	Lack of staff Lack of a team-based approach to care and interventions Lack of support from management Concern of harming the patient Concern of detachment of attached tubes Concern of side effects
Hassan and Wahsheh (2017) Jordan	To identify reasons for not applying VAP prevention measures	428	Quantitative cross-sectional design Descriptive survey Self-administered questionnaire	Lack of time No followed protocols in the units Patient discomfort Disagreement with guidelines
Jansson *et al.* (2013) Finland	To explore barriers towards VAP prevention EBGs	101	Quantitative cross-sectional design Descriptive survey Self-reported questionnaire	Inadequate resources Lack of time Lack of skills Lack of knowledge Lack of guidance
Jansson *et al.* (2018) Finland	To evaluate barriers to institution-specific ventilator bundle	108	Quantitative cross-sectional design Self-administered questionnaire	Role ambiguities Lack of education Inadequate resources Lack of outcome expectancy Patient discomfort Fear of adverse effects Disagreement Lack of guidelines Forgetfulness
Kiyoshi-Teo *et al.* (2014) USA	To identify factors influencing VAP prevention guidelines adherence	576	Quantitative cross-sectional design Descriptive survey Self-administered questionnaire	Time to actually complete the intervention
Ricart *et al.* (2003) Spain	To review barriers to nursing adherence to nonpharmacological EBGs for preventing VAP	51	Quantitative cross-sectional design Descriptive survey Self-administered questionnaire	Unavailability of resources (37.0%) Patient discomfort Fear of potential adverse effects Overwork or lack of time Costs
Yaseen and Salameh (2015) Saudi Arabia	To estimate Saudi critical care nurses’ knowledge regarding VAP prevention guidelines and explore the barriers that may restrict adherence	93	Quantitative cross-sectional design Descriptive survey	Lack of VAP courses Nursing shortage Lack of VAP knowledge during nursing education Lack of time
Yeganeh *et al.* (2019) Iran	To investigate barriers to implementing VAP prevention EBGs	219	Quantitative cross-sectional design Observation Questionnaire	Insufficient supply and equipment Insufficient educational seminars and materials

### Data synthesis

The relevant literature was summarized in separate tables based on the review aims. For this review, a critical appraisal of individual sources of evidence was not necessary ( Peters *et al.*, 2020). All authors independently reviewed the studies of NCBs in ICUs and extracted the barriers described in the results sections of the included studies. They were subsequently grouped into categories and subcategories, based on the identified contexts of barriers, using basic descriptive content analysis, which is an optimal method to summarize findings ( Elo & Kyngäs, 2008). The principles of inductive content analysis were used to analyze, categorize, and quantify NCBs for VAP prevention. After an initial open-coded base, similar open-codes were grouped together into thematic categories, each of which was labelled using content-specific keywords and subcategories.

## Results

**Figure 1.**
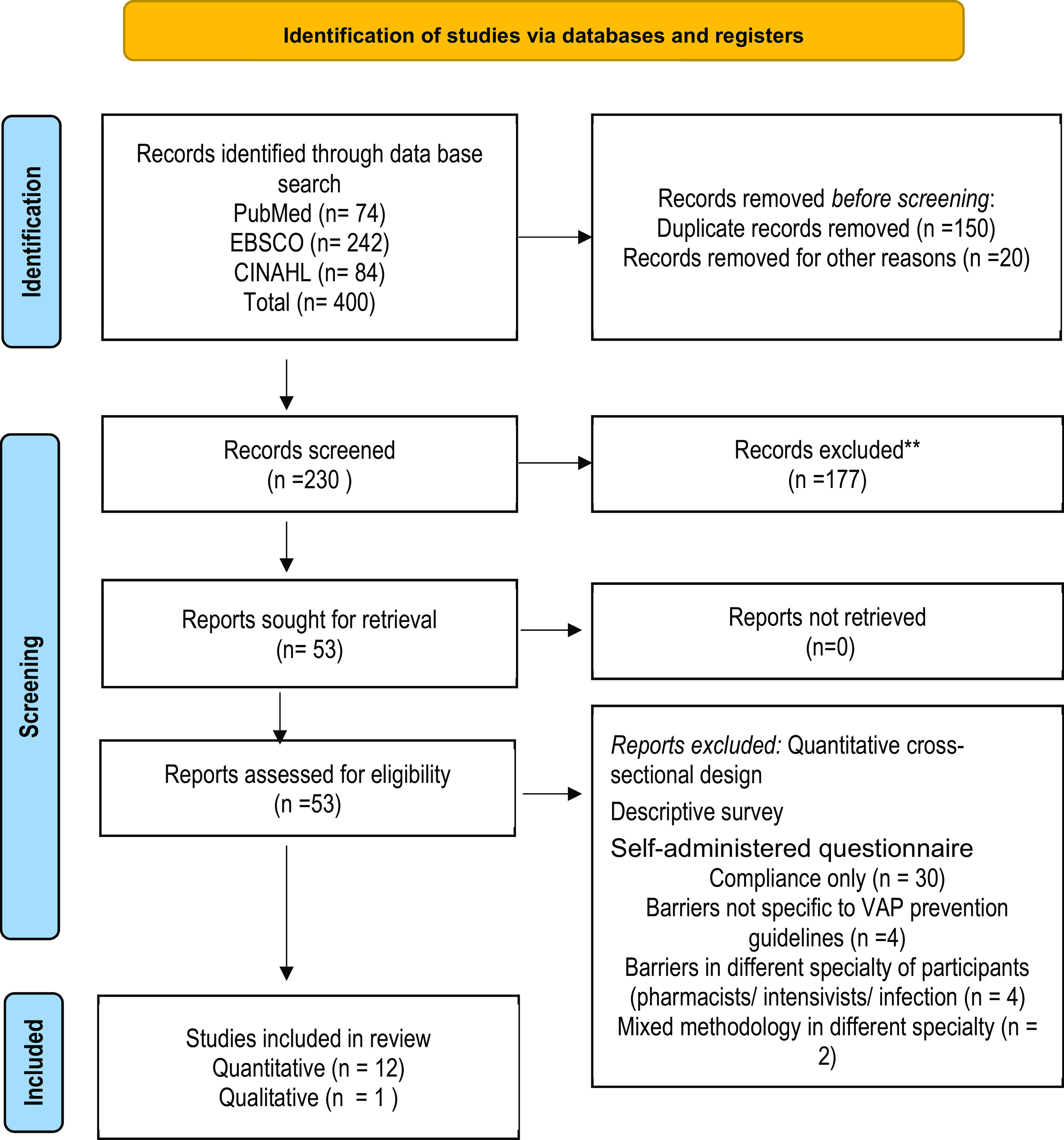
PRISMA flow diagram.

The initial search of this review identified 400 articles. A total of 230 non-duplicate records were subsequently identified through the searching of databases and the reference lists of included articles. After screening titles and abstracts (objectives of the articles) for relevancy against inclusion criteria, 53 articles were identified and retrieved for full-text data extraction and screening. Of these, 53 articles, 13 met all inclusion criteria and were included in this review. The flow chart ( Figure 1) of the review decision process was adapted from the PRISMA flowchart ( Page *et al.*, 2021).

### General characteristics of included studies

Of the 13 included studies (as shown in Table 3), one was qualitative ( Atashi *et al.*, 2018) and the remainder quantitative. The included studies were published between 2003 and 2022, with the following numbers of study per country: two (15.4%) in Finland ( Jansson *et al.*, 2013, 2018); three (23%) in Iran ( Atashi *et al.*, 2018; Dehghan *et al.*, 2022; Yeganeh *et al.*, 2019); two (15.4%) in Jordan ( Aloush & Al-Rawajfa, 2020; Hassan & Wahsheh, 2017); one (7.7%) in Jordan, Egypt, and Saudi Arabia (“Middle East”) ( Aloush *et al.*, 2018); one (7.7%) in Spain ( Ricart *et al.*, 2003); two (15.4%) in Saudi Arabia ( Al-Sayaghi, 2021; Yaseen & Salameh, 2015); one (7.7%) in Tanzania ( Bankanie *et al.*, 2021); and one (7.7%) in the USA ( Kiyoshi-Teo *et al.*, 2014).

**Table 3.** Summary of general characteristics of studies.

Study	Country	Aim/purpose	Methodology
Aloush *et al.* (2018)	Middle East (Jordan, Egypt, and Saudi Arabia)	To investigate the barriers and factors that affect nurses’ level of compliance with VAP prevention guidelines	Quantitative self-reported survey, cross-sectional design
Aloush & Al-Rawajfa (2020)	Jordan	To evaluate the compliance of Jordanian nurses with VAP prevention guidelines and barriers to compliance	Quantitative descriptive survey, self-administered questionnaire, cross-sectional design
Al-Sayaghi (2021)	Saudi Arabia	To identified critical care nurses’ compliance and barriers toward ventilator-associated pneumonia prevention guidelines	Quantitative descriptive survey self-reported questionnaire, cross-sectional design
Atashi *et al.* (2018)	Iran	To explore the perspectives of Iranian critical care nurses on the barriers to VAP prevention in ICUs	Qualitative descriptive design, semi-structured interviews
Bankanie *et al.* (2021)	Tanzania	To explore ICU nurses’ knowledge, compliance, and barriers towards VAP prevention EBGs in Tanzania	Quantitative descriptive survey, self-administered questionnaire, cross-sectional design
Dehghan *et al.* (2022)	Iran	To examine registered nurses’ perceived barriers towards VAP prevention in southeast Iran	Quantitative descriptive survey, self-administered questionnaire, cross-sectional design
Hassan & Wahsheh (2017)	Jordan	To identify the level of nurses’ knowledge of VAP and prevention measures before and after an educational program in Jordan	Quantitative descriptive survey, self-administered questionnaire, cross-sectional design
Jansson *et al.* (2013)	Finland	To explore critical care nurses’ knowledge of adherence to and barriers towards VAP prevention EBGs	Quantitative descriptive survey, self-reported questionnaire, cross-sectional design
Jansson *et al.* (2018)	Finland	To evaluate critical care nurses’ knowledge of, adherence to, and barriers toward institution-specific ventilator bundle	Cross-sectional design, self-administered questionnaires
Kiyoshi-Teo *et al.* (2014)	USA	Identify factors that influence adherence to guidelines for prevention of VAP	Quantitative descriptive survey, self-administered questionnaire
Ricart *et al.* (2003)	Spain	Review barriers to nursing adherence to non-pharmacological EBGs for preventing VAP.	Quantitative descriptive questionnaire
Yaseen & Salameh (2015)	Saudi Arabia	Estimate Saudi critical care nurses’ knowledge regarding VAP (VAP) prevention guidelines and to explore the barriers that may restrict adherence to these guidelines	Quantitative descriptive cross-sectional design survey
Yeganeh *et al.* (2019, 2015)	Iran	To investigate the barriers to implementing EBGs for VAP prevention	Quantitative observation, questionnaire, cross-sectional design

### Findings of the included studies

This review yielded evidence for variety of NCBs, categorized into barriers relating to the “work-environment”, “nurse”, and “situation”. These barriers were further categorized into subcategories of barriers: work environment ( *lack of equipment and supplies*; *lack of staff and time*; *lack of educational support*; and *ineffective supportive system*), nurse ( *limited personal competencies*), and situation ( *patient health, discomfort, and adverse events*) ( Table 4).

**Table 4.** Summary of key barriers identified (categories and subcategories).

Subcategories	Authors
**Work environment barriers**	
Lack of equipment and supplies	Atashi *et al.* (2018), Aloush and Al-Rawajfa (2020), Jansson *et al.* (2013), Yeganeh *et al.* (2019)
Lack of staff	Atashi *et al.* (2018), Al-Sayaghi (2021), Aloush and Al-Rawajfa (2020), Bankanie *et al.* (2021), Yaseen and Salameh (2015), Dehghan *et al.* (2022) Jansson *et al.* (2013) Ricart *et al.* (2003)
Lack of time	Al-Sayaghi (2021), Hassan and Wahsheh (2017). Jansson *et al.* (2013), Kiyoshi-Teo *et al.* (2014), Atashi *et al.* (2018), Yaseen and Salameh (2015), Ricart *et al.* (2003)
Lack of educational support	Aloush *et al.* (2018), Aloush and Al-Rawajfa (2020), Al-Sayaghi (2021), Atashi *et al.* (2018), Bankanie *et al.* (2021), Hassan and Wahsheh (2017), Jansson *et al.* (2013), Jansson *et al.* (2018), Yaseen and Salameh (2015), Yeganeh *et al.* (2019)
Ineffective supportive system	Aloush *et al.* (2018), Atashi *et al.* (2018), Bankanie *et al.* (2021), Dehghan *et al.* (2022), Jansson *et al.* (2018)
**Nurse barriers**	
Limited personal competencies	Aloush *et al.* (2018), Aloush and Al-Rawajfa (2020), Bankanie *et al.* (2021), Jansson *et al.* (2013), Jansson *et al.* (2018), Yaseen and Salameh (2015)
**Situational barriers**	
Patient health, discomfort, and adverse events	Al-Sayaghi (2021), Dehghan *et al.* (2022), Hassan and Wahsheh (2017), Jansson *et al.* (2018), Ricart *et al.* (2003)

## Discussion

### Overview

This review aimed to examine the characteristics and conclusions of studies to identify barriers confronting nurses, thwarting their compliance with EBGs for VAP prevention in ICUs. It is apparent that there has been an increased rate of publication of studies assessing NCBs over the past five years (during which nine out of the 13 identified studies since 2003 were published), which reflects the increasing significance attached to this issue in nursing clinical practice and research. It underscores the need to work resolutely in reducing VAP and improving QoC for MVPs in critical care settings, particularly in Middle Eastern countries, where six of the studies were conducted. Regarding the geographical distribution of the studies, one study each (7.7%) was conducted in North America and Africa, three (23%) in Europe, and eight (61.5%) in Asia. Evidently there have been few published studies aiming to explore barriers influencing nurses’ success of VAP prevention strategies implementation at Intensive care units.

Furthermore, the revised articles indicate a large degree of methodological homogeneity, with minimal variety in methodological approaches to explore barriers. The majority of the authors in the reviewed studies used quantitative approaches using self-reported questionnaires as the main data collection tool, with the exception of the study by Yeganeh *et al.* (2019), which used quantitative observational method to determine barriers; and that of Atashi *et al.* (2018), which used qualitative descriptive study with semi-structured interviews to allow a more in-depth understanding detecting real barriers experienced by nurses in ICUs.

Nurses in ICUs experienced various barriers that hinder their ability to implement the recommended VAP measures in a constant and consistent manner ( Atashi *et al.*, 2018). From the results of this scoping review, it is apparent that there are many complex, diverse and interconnected barriers that can prevent CCNs from being committed to and compliant with VAP PGs, which accordingly undermines QoC and exposes patients to VAP risk.

### Work environment-related barriers

Barriers related to the work environment are those beyond the control of individual nurse ( Jansson *et al.*, 2018). These include significant challenges in the workforce that hinder nurses from complying with VAP prevention EBGs. This review showed many barriers within work-environment context playing the paramount role in negatively affecting NC, diverting nurses from being compliant with VAP PGs.

### Lack of equipment and supplies

While medical supplies and equipment in ICU are vital interventional tools for VAP prevention, nurses widely reported a lack of endotracheal tubes with suctioning system and kinetic beds ( Aloush et al., 2018; Aloush & Al-Rawajfa, 2020; Jansson *et al.*, 2013; Yeganeh *et al.*, 2019), as well as deficiencies of personal protective equipment like gloves and face masks ( Aloush & Al-Rawajfa, 2020), which prevented them from applying appropriate VAP prevention. Yeganeh *et al.* (2019) observed the unavailability of most essential resources for VAP prevention (including endotracheal tubes with subglottic suctioning, closed suction systems, kinetic beds, etc.), comprising an insurmountable everyday obstacle to nurses that consistently and systematically prevented them from performing important VAP prevention strategies.

According to the nurses in Atashi *et al.* (2018) study, the QoC they delivered to prevent VAP was undermined because they lacked most prerequisite equipment stipulated and necessary in standard guidelines. Moreover, they were unable to prevent VAP because the physical structure of ICU did not include the required materials or permit required activities. For instance, the study reported that there was just one corridor available for the transport of both contaminated and clean materials, suctioning bottles were emptied into toilets, and there were minimal sinks available in the unit for hand washing. The nurses have no control over this problem, because the lack of required resources was due to cost control within the hospital management system. Atashi *et al.* (2018) concluded that medical supplies and equipment are essential to provide safe and high-QoC for patients, and hospitals’ cost control policies can negatively influence implementation of guidelines essential for VAP prevention, counter-productively increasing the cost of care over the long term ( Al-Sayaghi, 2021).

### Lack of staff and time

In ICU environments, nursing staffing is essential for providing continuous clinical services and improving the QoC of critically ill patients. Staff shortages result in overwork, burnout, and stress among working nurses, reducing nurses’ precision at work and causing the de-prioritization of VAP preventive measures amid a general reduction of QoC ( Atashi *et al.*, 2018). There was widespread consensus in the reviewed studies that a lack of staff, which can also be conceptualized as a low nurse-to-patient ratio or high demand, is a common NCB in ICUs at different levels ( Al-Sayaghi, 2021; Aloush & Al-Rawajfa, 2020; Atashi *et al.*, 2018; Bankanie *et al.*, 2021; Dehghan *et al.*, 2022; Yaseen & Salameh, 2015).

The most common barrier to implementing VAP prevention strategies reported by nurses in the studies by Al-Sayaghi (2021) and Dehghan *et al.* (2022) were the shortage of nursing staff, and these authors argued that inappropriate staff planning is a systemic issue that need to be addressed. Diminution of staff number in the ICU because of cost control policies minimizes the time available for nurses to provide appropriate VAP prevention procedures (to say nothing of holistic and non-biomedical care services, which are utterly disregarded in most busy and under-resourced ICUs). Similarly, Bankanie *et al.* (2021) concluded that almost all nurses experienced difficulty in adhering with appropriate VAP prevention strategies, despite their knowledge of such guidelines. This indicates that nurses feel overstretched doing many tasks simultaneously, struggling to deliver effective care to patients while juggling numerous patient- and system-related priorities.

The application of cost control policies results in a reduction of the number of nurses in ICUs, resulting in a reduction in procedure times ( Al-Sayaghi, 2021). When nurses work in units with lower bed capacities and lower workloads, they are more likely to comply with policies and guidelines, provide better patient care, and minimize infections ( Al-Sayaghi, 2021; Aloush *et al.*, 2018; Aloush & Al-Rawajfa, 2020; Dehghan *et al.*, 2022). This is because appropriate staffing numbers facilitate more time available for the nurses to provide the care required. Time constraints reduce QoC in clinical practice in numerous ways, which in ICUs mainly relate to the absolute prioritization of immediate biomedical needs, and a commensurate de-prioritization of long-term patient needs such as infection prevention and holistic dimensions of care. Many studies indicated that the limited time that nurses have to perform procedures related to VAP prevention is a significant factor in low NC with procedures needed for VAP prevention ( Al-Sayaghi, 2021; Hassan & Wahsheh, 2017; Jansson *et al.*, 2013; Kiyoshi-Teo *et al.*, 2014; Ricart *et al.*, 2003; Yaseen & Salameh, 2015).

Understaffing in ICUs engenders the lack of time that nurses face, and which contributes to their low NC ( Al-Sayaghi, 2021; Yaseen & Salameh, 2015). As a result of cost control policies, the number of nurses in the ICU decreases, making it difficult for procedures to be performed. Guidelines are more likely to be followed by nurses working in units with a lower workload. In an ICU with more nurses, NC would likely be better, and QoC would likely be improved ( Al-Sayaghi, 2021). While there was consensus that a lack of time affected the delivery appropriate guidelines for VAP prevention, the reasons to which this barrier were attributed differed among studies. Although common, it was not considered to be one of the main barriers to restrict adherence with guidelines in the study by Yaseen and Salameh (2015). Jansson *et al.* (2018) noted the very low significance of lack of time on low NC, and reported no effect of staffing levels on NC. Furthermore, a lack of time is not necessarily because of staff shortages. The nature of interventions in ICU also play an important role in maintaining a constant ambience of high-pressure demand and critical workload, which can contribute to the de-prioritization of non-immediate care needs.

However, other studies strongly emphasized the role of a lack of time in itself. Kiyoshi-Teo *et al.* (2014) considered that the time to actually complete the intervention is the only crucial environmental context of NC. Most nurses struggled to finish certain procedures compared to others, and they believed that time availability is positively associated with NC, specifically concerning oral hygiene guidelines. Therefore, time is a limiting factor for NC to complete certain interventions. Similarly, Ricart *et al.* (2003) reported that some nurses felt overloaded and had no time to perform “hand-washing between patient contacts”; and Atashi *et al.* (2018) found that some nurses felt overloaded most of the time, with patient situations necessitating a need for frequent suctioning, rendering it difficult for them to frequently wear gloves for each suctioning, which was flagged as another barrier to effective VAP prevention in ICUs.

Moreover, most nurses in Hassan and Wahsheh (2017) study were overwhelmed with routine ICU procedures due to lack of time, which hindered the proper application of important VAP guideline procedures. While some studies tackle both lack of adequate staffing and lengthy time needed to complete certain procedures, it was not always clear if other reasons were instrumental to the theme of a lack of time. Factors such as “non-nurse tasks” and “the use of electronic documentation” might be predisposing barriers that contribute to losing time of nurses from performing appropriate VAP prevention performance in ICUs.

### Lack of educational resources

In the included studies of this review, lack of education and training for VAP PGs at hospital settings was brought up many times as NCB in ICUs ( Al-Sayaghi, 2021; Aloush *et al.*, 2018; Aloush & Al-Rawajfa, 2020; Atashi *et al.*, 2018; Jansson *et al.*, 2018; Yaseen & Salameh, 2015; Yeganeh *et al.*, 2019). In-service staff education and training respecting VAP prevention can improve nurses’ knowledge and skills and improve care quality. However, most nurses in the above reviewed articles referred to the inadequacy and the ineffectiveness of staff training programs. Many nurses reported that the education they had received in their clinical training was not consistent with the VAP PGs, and more than half of them reported that they never been educated on VAP management courses in their hospitals, and only a third had been educated in the field of mechanical ventilator management based on the VAP PGs ( Aloush & Al-Rawajfa, 2020). This finding is congruent with the study of Al-Sayaghi (2021), which reported that a third of nurses never received hospital-based education or training regarding VAP prevention strategies.

Yeganeh *et al.* (2019) observed compiled education and educational seminars about VAP PGs in only a few units, with minimal educational posters and pamphlets. Atashi *et al.* (2018) revealed that novice nurses did not obtain adequate VAP training, and consequently felt anxious when providing care to their patients after only a week of supervision under senior nurses in their units during their ICU orientation. This ostensibly indicates a lack of necessary basic training for VAP prevention in such hospitals. In addition, the findings of both observation and interviews indicated that most nurses enrolled in VAP PG programs just to get licenses for career development, without really attending or engaging with the learning process wholeheartedly, with a lack of assessment of program effectiveness for improving nurses’ performance ( Atashi *et al.*, 2018). This indicates that nurses are less likely to adhere to VAP guidelines because of a lack of training courses and effective, sustainable education programs ( Al-Sayaghi, 2021; Aloush *et al.*, 2018; Jansson *et al.*, 2018; Yaseen & Salameh, 2015). Moreover, Aloush *et al.* (2018) revealed practicing VAP prevention in hospital is not based on research, therefore forming an NCB. Knowledge transfer among nurses is hindered by an inability to translate research into practice ( Aloush *et al.*, 2018; Bankanie *et al.*, 2021), and poor information-sharing between them, which is reflected in poor education and continuing professional education among nurses, associated with low employee satisfaction and QoC delivery ( Bankanie *et al.*, 2021).

Availability of written policies and protocol of nursing care for VAP prevention for MVP is essential and effective in ICUs to enhance nurses’ adherence with recommended guidelines. Studies found that more than half of the nurses deemed a lack of VAP prevention policy and protocol in their facilities to be a major NCB ( Aloush & Al-Rawajfa, 2020; Atashi *et al.*, 2018; Hassan & Wahsheh, 2017; Yaseen & Salameh, 2015). Moreover, nurses in Atashi *et al.*’s (2018) study argued that irrelevant VAP prevention protocols and recommendations established in foreign countries are being applied in settings where they are not necessarily applicable, and they recommended developing new institutionally sensitive guidelines, considering the features of particular settings, such as the number of nurses in shifts, specifications, and equipment availability. On the other hand, Aloush *et al.* (2018) found that nursing practice in ICUs not being based on research findings ( *i.e.*, a lack of evidence-based practice) is an NCB, reflected in nurses’ lack of awareness of updated knowledge for VAP prevention. Therefore, action is needed by healthcare systems and nurse educators to impart up-to-date evidence-based care, particularly educational sessions to explain protocols and policies for VAP prevention based on evidence-based sources, to help familiarize and orient nurses in order to expedite application of guidelines in practice contexts.

In addition, healthcare systems need to assess and improve the knowledge and capabilities of nurse educators and senior nurses to enable the diffusion of required VAP prevention knowledge and practice, aside from making training and educational materials available. The supervision of nurses in ICUs regarding their skills is a significant component behind improvement of nurses performance, and a lack of supervision is a barrier commonly identified by nurses themselves ( Atashi *et al.*, 2018), and the related issue of the absence of guidance for achieving standard VAP prevention, which is another known NCB ( Aloush *et al.*, 2018; Jansson *et al.*, 2013). Such features could be due to related barriers such as lack of time for monitoring, lack of supervision-related training, and lack of knowledge and abilities for efficient supervision by senior nurses who can guide practice.

Atashi *et al.* (2018) identified ineffective supervision as a main barrier for effective VAP prevention. Supervisors need to perform many nonsupervisory duties, which prevent them from effectively performing their activities related to supervision. For instance, they were mostly involved in resolving interpersonal conflicts, and making necessary adjustments for patient transfers to other hospitals. Managing educational resources involves the coordination of human and material resources to monitor, plan, strategize, and implement the delivery of education. Based on the reviewed articles, inadequate and inefficient staff training and education at clinical settings suggest the need of hospitals to adhere with the CDC’s 2003 recommendations on the importance of conducting education and training programs of nurses in ICUs ( Tablan *et al.*, 2003), for effective implementation of VAP prevention strategies.

### Ineffective supportive system

Related to the resource constraints described previously, which are related to hospital management, management’s direct support for senior nurses, including managers and supervisors, is also essential for the success of VAP implantation. Four reviewed studies found that insufficient management support is an NCB ( Aloush *et al.*, 2018; Atashi *et al.*, 2018; Bankanie *et al.*, 2021; Dehghan *et al.*, 2022; Jansson *et al.*, 2018). Passive and ineffective management affects the accuracy in task performance of nurses, forming an NCB ( Atashi *et al.*, 2018). Other barriers than nurses believed as barriers include limited professional competence, low job motivation, and limited professional accountability, all of which reflect organizational and systemic issues. Nursing managers need to understand how the scarcity of hierarchical support and passive human resource management have impacts on the implementation success of guidelines.

Jansson *et al.* (2018) reported that passive management was instrumental in nurses’ ambiguous perceptions of their role in VAP prevention strategies. Role ambiguity was identified as a barrier because nurses are uncertain about their definite tasks in implementing VAP prevention. A lack of clarity about expected roles may cause nurses to struggle, despite their intrinsic role in the evidence-based practice paradigm, the policies of individual hospitals, and of health systems in general, may not be clear for nurses ( *e.g.*, whether sedation interruption is a nursing task, or whether only physicians have this authority). Hospital management needs to provide more support by clarifying and setting clear rules and responsibilities for nurses in implementing strategies.

Moreover most of the nurses in Bankanie *et al.*’s (2021) study noted low “job discretion” to be a barrier. Nurses are not allowed to make responsible choices, judgments, or decisions with their patients in ICU. This might be as a result of unsupportive system along with job ambiguity in addition to lack of knowledge or skills. Nurses in the study of Dehghan *et al.* (2022) indicated that a lack of managerial support within the hospitals greatly undermines NC, which is related directly to healthcare organizational systems. These barriers reflect the low quality of working life, and poor organizational culture, which directly influence nurses’ satisfaction and their readiness to deliver higher QoC.

The work environment also includes social features within team that can impact workplace relationships, collaboration, efficiency at work, which have impacts on NC. Nurses are the most vital members within healthcare teams, and their role as patient advocates and holistic care specialists is paramount in managing VAP impacts on ICU patients. In this role, nurses must work with each other as peers, and with other healthcare professions such as intensivists and respirologists, to prevent VAP in ICUs. Based on the findings within the review, nurses may be affected by peer influence and teamwork issues.

Issues of peers and teams significantly influence nursing performance, and adherence with guidelines, which also intersects with the issue of role models; nurses revealed that the lack of professional role models during their working activities comprises an NCB ( Aloush *et al.*, 2018). Nurses mentioned that the lack of a team-based approach to care and interventions is a big NCB, which might be due to severe shortages ( Dehghan *et al.*, 2022). In their study, Atashi *et al.* (2018) found that colleagues’ negative professional attitudes inhibit accurate VAP prevention practice. Participants’ reports indicated that some colleagues failed to perform their work accurately. They sometimes documented that they performed procedures for VAP prevention when they really did not, such as documenting endotracheal tube cuff pressure without actually measuring it.

### Nurse-related barriers

These barriers are nurse-related characteristics that limit the nurse’s ability to follow the guidelines ( Aloush *et al.*, 2018; Yaseen & Salameh, 2015). In this review nurse-related barriers included issues related to limited personal competences ( *e.g.*, education and knowledge, skills, and experience), and situation-related barriers ( *e.g.*, concerning the ICU clinical context), which pertain to the essential prerequisites for the success of VAP prevention in ICUs.

Based on the reviewed articles, personal characteristics may be instrumental in NC. The findings showed a lack of NC might be entrenched in nursing education. Aloush *et al.* (2018) reported that 63% of nurses in their study had received no education about VAP PGs in their nursing education, and the low level of VAP-related education among ICU nurses directly undermined their NC. They also revealed that there was a statistically significant difference based on academic degree, whereby nurses with masters’ degrees had higher NC in comparison with those with baccalaureate and diploma degrees, which was later affirmed by Bankanie *et al.* (2021). Aloush and Al-Rawajfa (2020) reported that the education nurses received during their study was not consistent with VAP PGs, adding a further barrier to their NC. It is important for schools of nursing to consider improving educational programs to improve nurses’ ICU knowledge and VAP-related education, consistent with clinical guidelines, to subsequently enhance nurses’ NC and QoC when they transition to clinical practice ( Aloush *et al.*, 2018; Aloush & Al-Rawajfa, 2020; Yaseen & Salameh, 2015).

Some studies reported a lack of skills to be an NCB. Jansson *et al.* (2013) and Bankanie *et al.* (2021) stated that a lack of skills among nurses was a significant barrier to compliance with guidelines for VAP prevention, and they called for ongoing educational interventions and effective strategies to facilitate knowledge and skills dissemination and transfer in the workplace. Almost a quarter (23%) of nurses “strongly disagreed” that a lack of skills was considered a barrier in a study by Aloush and Al-Rawajfa (2020), but this may reflect social desirability bias ( *i.e.*, nurses not wishing to acknowledge to others or to themselves that they lacked the required skills for serious interventions due to perceiving this to be a personal rather than systemic and educational shortcoming). Aloush *et al.* (2018) reported that years of experience was a significant indicator for increased NC, and numerous other studies found that less experienced nurses had lower NC in Middle Eastern contexts ( Aloush & Al-Rawajfa, 2020; Yaseen & Salameh, 2015); conversely, Jansson *et al.* (2013, 2018) found no relation between NC and longer work experience in Finland.

### Situation-related barriers

Situation-related barriers are factors related to patients that hinder nurses’ compliance toward VAP guidelines ( Jansson *et al.*, 2018; Ricart *et al.*, 2003) and to doubts that nurses have that can influence nurses’ behavior toward these guidelines ( Dehghan *et al.*, 2022; Jansson *et al.*, 2018). Some nurses questioned the importance of VAP prevention measures to patient well-being because, in certain clinical situations, they were required to make appropriate judgments and timely decisions when encountering patients with serious physiological issues. The reason for these concerns could be that certain clinical guidelines do not adequately consider individual patient needs and capabilities ( Jansson *et al.*, 2018). Unpredictable adverse effects harming patients, and undesirable patient outcomes from some VAP preventive procedures, were cited as dreaded outcomes by nurses in many studies ( Al-Sayaghi, 2021; Dehghan *et al.*, 2022; Hassan & Wahsheh, 2017; Jansson *et al.*, 2018; Ricart *et al.*, 2003).

Jansson *et al.* (2018) reported that nurses’ concerns regarding the impacts of VAP prevention procedures may have hindered adherence with appropriate strategies. In particular, nurses were doubtful of the indications of sedation, and worried about over-sedation that might harm patients. They also were worried about potential mistakes due to keeping their patients in a semi-recumbent position, as it is difficult to estimate the appropriate angle of the head of the bed. Uncertainty about indications of enhanced oral care, the estimated depth during endotracheal suctioning, and the duration of suctioning added to their fear of potential complications. Other studies reported that nurses were commonly concerned about the detachment of attached tubes during certain VAP prevention procedures ( Dehghan *et al.*, 2022). These concerns might be because of the fear of committing mistakes *per se*; producing undesired alterations in hemodynamic status of patients; or the belief that performing VAP measures could cause deterioration in the critical status of patients. These concerns made the nurses more cognizant of their patients’ holistic needs, and they experienced a dilemma between the biomedical mandates of VAP prevention and what they perceived to be their nursing duty of safeguarding patients holistically, which resulted in noncompliance with some VAP preventive measures.

## Conclusion

Common barriers appear to inhibit nurses from performing appropriate VAP prevention strategies. This review highlighted the intricate correlated barriers that inhibit NC with evidence-based VAP prevention strategies. The aim of this review to identify gap in literature to guide future research. The ICU work environment and hospital management play major roles in creating low NC, without appropriate measures to address nursing and situational impediments to compliance, such as the effects of attitudes and behaviors, efficacy, low job motivation, peer influence and team dynamics, all of which need further scrutiny and clarification. Contextual and work environment barriers are relatively under-reported, and warrant further exploration, but it is clear that the work environment is the base issue that triggers multiple NCBs, beyond the control of individual nurses.

This scoping review can be used as a template for future studies, representing the key concepts underpinning NCBs among nurses in intensive care units. Further primary research about barriers using different methodological approaches, such as mixed-methods studies and more exploratory qualitative studies can gain more in-depth insights and find out the thorough predisposing barriers formulating a solid literature. Comparative studies of hospitals with zero VAP rate and high VAP rate within the same and/or other countries are essential to solidly elucidate real barriers. Follow-up studies to identify the barriers, plan and then implement changes to improve NC, and monitor related KPIs (particularly VAP rate) would clarify systemic and long-term barriers more fully. Such research may help to solidly elucidate all barriers that might be essential for nurse leaders, and policymakers particularly if various types of study are conducted within the same local context. It would be beneficial to support nursing leaders and healthcare systems in augmenting compliance and informing practice, positively influencing QoC for MVPs, and empowering nurses to able to identify and control their own barriers.

### Strengths and limitations

The duration of the search encompassed works published since 2003, which was included all recent works published in English. The numerous specializations involved in intensive care units allow generalization of the findings of this study for all CCNs, in terms of barriers hindering nursing compliance with VAP prevention strategies. Moreover, this is the first scoping review that considered a standalone study for future research using a rigorous PRISMA-ScR report, adding more strength to the review.

However, this scoping review also has some limitations. To make it more feasible, this review included only published, peer reviewed research articles in English language, available in full-text form. These criteria may have led to missing some relevant studies and information. Future reviews may further include other types of literature, such as grey literature, reports, dissertations, and editorials, and works in non-English languages. Future reviews in this topic may compare published findings with those of unpublished literature.

## Data Availability

Figshare: Barriers to compliance with evidence-based guidelines for ventilator-associated pneumonia among critical care nurses: A scoping review, https://doi.org/10.6084/m9.figshare.21493122.v1 ( Al-Tamimi *et al*., 2022). The project contains the following underlying data: Search Strategy. File 1- PubMed search strategy performed June 13, 2022 Repository name: Figshare: https://doi.org/10.6084/m9.figshare.21493122.v1 ( Al-Tamimi *et al*., 2022). This project contains the following extended data:
• Data extraction form. Summary review of included studies) Figshare: PRISMA-Scr checklist for ‘Barriers to compliance with evidence-based guidelines for ventilator-associated pneumonia among critical care nurses: A scoping review’, https://doi.org/10.6084/m9.figshare.21493122.v1 ( Al-Tamimi *et al*., 2022). Data are available under the terms of the Creative Commons Zero “No rights reserved” data waiver (CC0 1.0 Public domain dedication).
