# Supplementary material for: Barriers to compliance with evidence-based guidelines for ventilator-associated pneumonia among critical care nurses: A scoping review
Source: F1000Res. 2023 Mar 24;11:1551. Originally published 2022 Dec 21. [Version 2] doi: 10.12688/f1000research.128144.2 (PMC10076907; doi:10.12688/f1000research.128144.2)
Supplement: Peer review history

# Peer review: Reviewer response for version 2

**DOI:** 10.5256/f1000research.145714.r167703

**Published:** 2023-04-05

**Authors:** Khaled Mohammed Al-Sayaghi

**Affiliations:** 1 Critical Care and Emergency Nursing, College of Nursing, Taibah University, Madinah, Saudi Arabia

I appreciate the authors response to comments, the paper in its current condition became more suitable for indexing. I approve it.

Are the rationale for, and objectives of, the Systematic Review clearly stated?

Yes

Is the statistical analysis and its interpretation appropriate?

Yes

Are sufficient details of the methods and analysis provided to allow replication by others?

Yes

Are the conclusions drawn adequately supported by the results presented in the review?

Yes

Reviewer Expertise:

Critical Care Nursing, infection control practices, nutritional support in critical care, and mechanical ventilation

I confirm that I have read this submission and believe that I have an appropriate level of expertise to confirm that it is of an acceptable scientific standard.

# Peer review: Reviewer response for version 2

**DOI:** 10.5256/f1000research.145714.r167704

**Published:** 2023-03-30

**Authors:** Intima Alrimawi

**Affiliations:** 1 Nursing, Georgetown University, Washington, District of Columbia, USA

I am happy with the changes, and I do approve the current version of the study for indexing.

Are the rationale for, and objectives of, the Systematic Review clearly stated?

Yes

Is the statistical analysis and its interpretation appropriate?

Not applicable

Are sufficient details of the methods and analysis provided to allow replication by others?

Yes

Are the conclusions drawn adequately supported by the results presented in the review?

Yes

Reviewer Expertise:

Her research focuses on improving the quality of care for vulnerable families and children with complex health concerns and critical illness and developing nursing education, mainly in low and middle-income countries.

I confirm that I have read this submission and believe that I have an appropriate level of expertise to confirm that it is of an acceptable scientific standard.

# Peer review: Reviewer response for version 1

**DOI:** 10.5256/f1000research.140706.r158511

**Published:** 2023-01-16

**Authors:** Intima Alrimawi

**Affiliations:** 1 Nursing, Georgetown University, Washington, District of Columbia, USA

This scoping review discusses the topic of barriers to compliance with evidence-based guidelines for ventilator-associated pneumonia among critical care nurses. This is a very important topic that was not covered in other scoping reviews. 

 In the methodology section the researcher thoroughly discussed the scientific approach that they followed to conduct this review. 

 The results section needs to be developed further; additional discussion is needed around the selected studies and the final themes that did emerge from them.

 The discussion section needs to take the themes to the next level and discuss the potential implication and recommendations to recommend these barriers. For example, if you think that lack of staff and time was a barrier, then what is the implication of this in the current practice, and how can we overcome this barrier on the ground? 

 The conclusion section is long, it needs to be summarized and be more focused.

 You can also add a separate recommendation section. 

Are the rationale for, and objectives of, the Systematic Review clearly stated?

Yes

Is the statistical analysis and its interpretation appropriate?

Not applicable

Are sufficient details of the methods and analysis provided to allow replication by others?

Yes

Are the conclusions drawn adequately supported by the results presented in the review?

Yes

Reviewer Expertise:

Her research focuses on improving the quality of care for vulnerable families and children with complex health concerns and critical illness and developing nursing education, mainly in low and middle-income countries.

I confirm that I have read this submission and believe that I have an appropriate level of expertise to confirm that it is of an acceptable scientific standard.

# Peer review: Reviewer response for version 1

**DOI:** 10.5256/f1000research.140706.r158512

**Published:** 2023-01-16

**Authors:** Khaled Mohammed Al-Sayaghi

**Affiliations:** 1 Critical Care and Emergency Nursing, College of Nursing, Taibah University, Madinah, Saudi Arabia

Overall the review is so good. It provides information about the barriers to compliance with evidence-based guidelines for VAP among critical care nurses. It considered one of the important topics in the critical care nursing. I thank the authors for choosing this important topic. Kindly consider the following comments:

1. The small paragraphs in the introduction can be merged into 4 large paragraphs.
2. Has the systematic review been registered in specific institutions such as PROSPERO?
3. When conducting a systematic review, it is normal to provide some additional information on the search methodology (search strategy).
4. Regarding the search strategy, I see that synonyms of the keywords for the research topic can be helpful. Synonyms provide access to all the research published on the subject of the research, for example, barriers may use factors, nurse may use health care workers, guideline may use bundle, compliance may use adherence, intensive care may use critical care, etc. The use of the single term is likely to miss quite a lot of the literature on this subject.
5. Is it possible to pool the effect size of each compliance barriers among critical care nurses by meta-analysis? If not, please explain the reasons.
6. The researchers in the discussion did not talk about the secondary research that dealt with the subject in some detail and how this study differs from previous reviews.
7. You need to provide operational definitions for each barrier you mentioned in the results.
8. The work environment-related barriers were categorized into several subcategories. If possible, categorize the “Nurse-related barriers” and “Situation-related barriers”.
9. Page 13, line 11: “Limited personal competencies” is the only subcategory under the “Nurse-related barriers”. If it is not possible to add other subcategories, it is better to remove the subtitle.
10. Page 14: there is a redundancy in the second and third paragraphs of conclusions. It needs to be summarized in one small paragraph.
11. You need to add a subheading about implications for nursing.
12. The paper needs professional English editing.

Are the rationale for, and objectives of, the Systematic Review clearly stated?

Yes

Is the statistical analysis and its interpretation appropriate?

Yes

Are sufficient details of the methods and analysis provided to allow replication by others?

Yes

Are the conclusions drawn adequately supported by the results presented in the review?

Yes

Reviewer Expertise:

Critical Care Nursing, infection control practices, nutritional support in critical care, and mechanical ventilation

I confirm that I have read this submission and believe that I have an appropriate level of expertise to confirm that it is of an acceptable scientific standard, however I have significant reservations, as outlined above.
